# Analysis of risk factors for difficult implant removal in children with slipped capital femoral epiphysis treated by cannulated screws

**DOI:** 10.3389/fped.2024.1414557

**Published:** 2024-05-22

**Authors:** Lei Yang, Lijun Liu, Xiaodong Yang, Xueyang Tang

**Affiliations:** West China Hospital, Sichuan University, Chengdu, China

**Keywords:** slipped capital femoral epiphysis (SCFE), cannulated screws, difficult implant removal, risk factors, pediatric

## Abstract

**Introduction:**

Cannulated screws are widely used in the treatment of slipped capital femoral epiphysis, which can be removed after physeal closure on patient's request. This study aimed to analysis the potential risk factors for difficult removal in children with slipped capital femoral epiphysis treated by cannulated screws.

**Patients and methods:**

This study enrolled 32 hips that had undergone removal of cannulated screws after treatment of slipped capital femoral epiphysis at our department. The primary outcomes were the difficult screw removal. The secondary outcomes were functional outcome assessed by using a modified Harris Hip Score and complications of fractures and surgical site infection. Related risk factors for difficult removal were recorded and analyzed by multivariable logistic regression.

**Results:**

In total, 32 hips were evaluated, with a mean age of 14.9 ± 1.3 years old (range, 13–19 years). Six (18.8%) hips presented with difficult removal, including 4 cases of screws’ slip and 2 breakages. The average implantation time in the difficult removal group (5.7 ± 1.0) was also significantly longer than that in the easily removed group (3.8 ± 0.9, *p *= 0.001). The mean surgical time in patients with difficult removal was 66.3 ± 11.6 min, which was also significantly longer than that (54.8 ± 8.3) in the other patients (*p *= 0.008). The duration of screw implantation was an independent risk factor for difficult removal.

**Conclusions:**

Prolonged screw duration was a predictor for difficult removal in children with slipped capital femoral epiphysis treated by cannulated screws. An early surgery after physeal closure might benefit those with a request for screw removal.

## Introduction

Slipped capital femoral epiphysis (SCFE) is a rare hip disorder that involves displacement of the proximal femoral metaphysis to the epiphysis in adolescent young patients (1, 2). The true incidence of SCFE varies greatly in different regions, but with an overall trend of increasing and a higher ratio of incidence in males (3). The treatment options include hip spica, bone graft epiphysiodesis, pinning *in situ*, closed/open reduction and fixation, Dunn osteotomy, Ganz surgical dislocation, and others (4, 5). Cannulated screws are widely used in the treatment of SCFE due to its precise guidance for screw implantation and minimally invasive procedure (6). Considering patients’ request for removal and the risk of complications for long-time screw implantation, screw removal after physeal closure is widely accepted by surgeons and patients (7, 8). While there seems to be a higher rate of removal failure for these screws in SCFE than other implantations (6, 9, 10), few studies focused on this topic.

Thus, this study aimed to describe the outcomes and complications of screw removal and analyze the potential risk factors for difficult removal in children with slipped capital femoral epiphysis treated by cannulated screws.

## Patients and methods

There was a total of 30 cases underwent screw removal at our department from January 2015 to January 2022. All these patients were previously diagnosed with SCFE and had undergone surgical treatment and fixation with the single cannulated screw. All screws used for SCFE treatment in our center were full-thread cannulated titanium screws. The implant removal after physeal closure was carried out for patients if requested, rather than for everyone. All the procedures were performed by two senior pediatric orthopedic surgeons well-trained in this technique and SCFE fixation. The inclusion criteria were as follows: patients treated at our center, with complete medical information and a minimum follow-up of 1 year. The exclusion criteria were patients with no intention of screw removal or being treated in outside facilities, incomplete medical information, or those with less than 1 year follow-up.

The baseline data were collected from the hospital records, including gender, side, age at SCFE fixation, age at removal, duration of screw implant, screw lengths, surgical time, and difficulty in removal. “Difficulty removal’’ was defined as screw's slip or breakage during removal with the normal screwdriver, and additional tools was required. For the broken screw, a guide wire was inserted through the cannulated screw, then reaming of femoral canal with a specially designed hollow drill, and finally the screw was extracted by using a T-handle bar. Surgery was performed by one senior pediatric orthopedic surgeon under general anesthesia. All patients were assessed functionally by another independent pediatric orthopedic. The primary outcome of this study was the rate of difficult removal and the risk factors for removal difficulty. The secondary outcomes were complications, surgical time, and functional results assessed by using the modified Harris Hip Score (mHHS) at the first-year follow-up.

SPSS 29 was used for data analysis. Continuous data were reported using the mean ± standard deviation (SD) and range. Categorical data are reported as numbers and percentages. Risk factors for difficulty removal were evaluated using a logistic regression model, and odds ratios (ORs) with 95% confidence intervals (CIs) were also obtained. Chi-squared tests and student’s *t*-tests were also used in a subgroup or univariate analysis. A *p*-value of less than 0.05 was considered significant.

## Results

**Figure 1:**
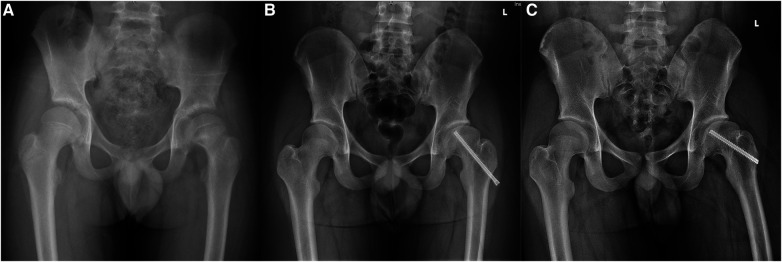
Radiographs of a 17-years old boy with difficult screw removal on the left side. (**A**) Pelvic radiograph of a 10-year-old boy with left slipped capital femoral epiphysis. (**B**) Preoperative pelvic radiograph of the patient at the age of 17 years old when planned to remove the screw. (**C**) Removal of the screw through an open approach was difficult and unsuccessful. The screw was not removed entirely.

In total, 32 hips (17 on the left side, 15 on the right side) were evaluated in our study, with a mean age of 14.9 ± 1.3 years old (range, 13–19 years). Six (18.8%) hips presented with difficult removal, including 4 cases of screws’ slip and 2 breakages. Except for one case of breakage that the parents did not request to attempt Figure 1, all other 5 difficult screws were ultimately removed with additional tools. The average implantation time in the difficult removal group (5.7 ± 1.0) was significantly longer than that in the easily removed group (3.8 ± 0.9, *p* = 0.001). The mean surgical time in patients with difficult removal was 66.3 ± 11.6 min, which was also significantly longer than that (54.8 ± 8.3) in the other patients (*p* = 0.008). There was no significant difference in the functional outcome between the difficult removal group (87.5 ± 10.4) and the easy removal group (88.7 ± 4.4, *p* = 0.799). No infection, wound-healing failure, anesthetic complications, nerve injury, fracture, or other complication was observed. The detailed data is presented in Table 1. The duration of screw implantation (OR, 4.44; CI, 1.29–15.22, *p* = 0.018) was an independent risk factor for difficult removal (Table 2).

**Table 1:** Patients’ characteristics and difficult removals.

Characteristics	Difficult implant removals (*n* = 6)	Easy implant removals (*n* = 26)	*P*
Gender			1.000
Female	2	10	
Male	4	16	
Side			0.383
Right	4	11	
Left	2	15	
Age at fixation, years	10.3 ± 1.6	10.9 ± 1.4	0.411
Age at removal, years	16.1 ± 2.2	14.7 ± 0.9	0.085
Duration of implants	5.7 ± 1.0	3.8 ± 0.9	**0** **.** **001**
Screw lengths	85.8 ± 8.0	83.7 ± 4.6	0.373
Removal surgical time	66.3 ± 11.6	54.8 ± 8.3	**0**.**008**
Functional outcomes	87.5 ± 10.4	88.7 ± 4.4	0.799

Bold values are statistically significant *p* < 0.05.

**Table 2:** Multivariate analysis for risk factors of difficult removals.

Characteristics	Risk factors	Odds ratio	95% CI	*P*
Difficult removals	Duration of implants	4.44	(1.29–15.22)	**0** **.** **018**
	Age at removal	1.83	(0.59–5.70)	0.297

CI, confidence interval. Bold values are statistically significant *p* < 0.05.

## Discussion

Though SCFE is a rare disease, it is one of the most common hip disorders among children aged 9–15 years old, with an average annual incidence of 4.4/10,000 for girls and 5.7/10,000 for boys (2, 11). Treatment options vary from single screw in-situ fixation to modified Dunn procedure, based on the severity of the deformity, classification, stability, age, and others (3, 5, 12). The common method for mild chronic SCFE is *in situ* fixation with a single screw through the femoral neck and into the epiphysis, to prevent further slip (12, 13). The number of screws needed to maintain stability and the choice of implant is controversial. Two or multiple screws fixation has also been reported in the literature, while it seems to cause more complications than a single screw (12, 14). The types of screws include fully threaded screws, partially threaded, and some other growth-facilitating screws intended to avoid growth arrest (15). And there are some types of screws specifically designed for SCFE fixation, which may be easier to remove. However, few studies have compared the long-term outcomes or hardware complications of these different types of implants (13). Usually, thin, or non-threaded implants should be avoided as there was a potential risk of bending or migration (16). For this group of patients, a standard full-thread single cannulated screw was used for the fixation surgery.

In most cases, implant removal is a selective orthopedic surgery. In China, patients usually have a strong desire to have their implants removed despite experiencing no pain or discomfort, which may be a result of the traditional culture or patient's request (17). Most implants can be easily removed, but there is still an overall complication rate of 10% in all removal surgeries, which is even higher in SCFE implant removal (6, 18). Younger age, longer time from fixation to removal, and type of implants have been identified as risk factors for difficult removal in many other removal surgeries (10). While few studies focused on the removal surgeries of treated SCFE in children.

Pretell-Mazzini et al. (6) found that the type of screw alone was a significant risk factor for difficult removal after SCFE fixation treatment. The use of full-threaded cannulated stainless-steel screws could decrease the removal failure risk. Vresilovic et al. (7) reported similar results that the pin type and size were significantly related to removal failure rate, and cannulated titanium or non-cannulated small pins were not recommended for treatment of SCFE. The possible reasons might be the better ingrowth behavior of titanium in the bone and its higher elasticity make it more difficult to remove. In our study, only one type of screw was applied, thus we could not draw any conclusion about the effect of screw type on difficult removal. That was also one of the limits of the study.

Duration of implantation can affect the difficulty of removal. Hou et al. (17) reported that a longer interval between fixation and removal could lead to difficulties in the removal of the locking compression plates and screws of both the upper and lower extremities. Lee et al. (19) described that the retrieval problems occurred with both stainless steel and titanium devices if implanted for more than 1 year. In the study by Pretell-Mazzini et al. (6), they found that the duration of screw implantation could prolong the surgical time with an increase of 16 min if the screw was implanted for more than 2 years. It was explained that the longer time of implantation, the harder bone/screw interface could be formed, which might lead to more difficulties for removal and more time for surgery. This was also consistent with our results that the difficult removal surgery did take more time than the easy removal surgery. Thus, many authors suggested that the removal surgery should be performed as soon as radiographs show fracture healing if removal is indicated or required.

For SCFE, screw removal should only be performed after physeal closure, which might take a long time and lead to an increase in implant duration (7, 8). In our study, the mean duration of implants was 5.7 ± 1.0 years in the difficult removal group, and 3.8 ± 0.9 years in the easily removed group (*P* = 0.001), which was longer than that in other studies. In addition to the long time required for physeal closure, another possible reason was poor patient compliance. In our study, implant removal after physeal closure was mainly based on the patient's request, which provided patients with a wide range of options for surgical timing and might lead to a prolonged duration of implant.

Difficult removal often means more attempts, additional procedures, and more removal devices, which might cause additional bone damage or more compilations (9). The primary risk of implant removal in children is refracture (20), others include the complications of soft tissue damage and infections (21). For this reason, some authors do not recommend unnecessary implant removal. In the study of screw removal of SCFE by Pretell-Mazzini et al. they described a very low incidence of removal complications, and no fracture or infection was observed (6). In our study, there was also no complication of fractures or surgical site infection. The only minor complication was failure to remove the screw in one case. The functional results at 1-year follow-up showed no significant difference between the difficult removal group and the easily removed group. The low incidence of complications reported in the literature, or our study might be due to the small sample size of the SCFE studies.

Other limitations of the study included retrospective, non-comparative study design, and lack of long-term follow-up. Another limitation was that we only used one type of screw in the treatment SCFE. Therefore, it was not possible to evaluate the impact of screw types on removal difficulty, which has been described as a risk factor for difficult removal in some studies. Further prospective research is needed to compare different techniques of screw insertion and direction to determine whether the direction of screw affects removal.

## Conclusions

Prolonged screw duration was a predictor for difficult removal in children with slipped capital femoral epiphysis treated by cannulated screws. An early surgery after physeal closure might benefit those with a request for screw removal. Once the removal is attempted, the patient and parents should be well informed about the possibility of difficulty or inability of the removal.

## Data Availability

The raw data supporting the conclusions of this article will be made available by the authors, without undue reservation.
